# Genetic Diversity and Population Structure of Myanmar Rice (*Oryza sativa* L.) Varieties Using DArTseq-Based SNP and SilicoDArT Markers

**DOI:** 10.3390/plants10122564

**Published:** 2021-11-24

**Authors:** Aye Aye Thant, Hein Zaw, Marie Kalousova, Rakesh Kumar Singh, Bohdan Lojka

**Affiliations:** 1 Department of Crop Sciences and Agroforestry, Faculty of Tropical AgriSciences, Czech University of Life Sciences Prague, Kamýcká 129, Praha 6 Suchdol, 165 00 Prague, Czech Republic; marie.kalousova@gmail.com; 2 Plant Biotechnology Center, Pale Myothit, Shwe Nanthar, Mingaladon, Yangon 110 23, Myanmar; heinzawagri@gmail.com; 3 International Center for Biosaline Agriculture, Crop Diversification and Genetics, Al Rwayyah 2, Academic City, Dubai P.O. Box 14660, United Arab Emirates; r.singh@biosaline.org.ae

**Keywords:** DArT markers, genetic diversity, Myanmar, rice (*Oryza sativa* L.), silicoDArT, SNP

## Abstract

Myanmar is well known as a primary center of plant genetic resources for rice. A considerable number of genetic diversity studies have been conducted in Myanmar using various DNA markers. However, this is the first report using DArTseq technology for exploring the genetic diversity of Myanmar rice. In our study, two ultra-high-throughput diversity array technology markers were employed to investigate the genetic diversity and population structure of local rice varieties in the Ayeyarwady delta, the major region of rice cultivation. The study was performed using 117 rice genotypes with 7643 SNP and 4064 silicoDArT markers derived from the DArT platform. Genetic variance among the genotypes ranged from 0 to 0.753 in SNPs, and from 0.001 to 0.954 in silicoDArT. Two distinct population groups were identified from SNP data analysis. Cluster analysis with both markers clearly separated traditional Pawsan varieties and modern high-yielding varieties. A significant divergence was found between populations according to the Fst values (0.737) obtained from the analysis of molecular variance, which revealed 74% genetic variation at the population level. These findings support rice researchers in identifying useful DNA polymorphisms in genes and pinpointing specific genes conferring desirable phenotypic traits for further genome-wide association studies and parental selection for recombination breeding to enhance rice varietal development and release.

## 1. Introduction

Rice (*Oryza sativa* L.) is one of the most widely cultivated cereal crops distributed across the world and serves as a major food source for more than half of the global human population [1]. Because of the rapid growth of the world’s population, as well as increasing urbanization and climatic changes, higher or at least stable rice yield is urgently required to meet world food demand [2]. Myanmar is the seventh-largest rice- producing country in the world, with a total rice production of 25.9 million tons in 2018 [3]. Rice is the main staple food crop in Myanmar, but is also cultivated as the most important cash crop for the majority of farmers, with large export potential. It is grown extensively across the whole country, covering an area of 7.26 million ha, and its annual production reaches 28.1 million tons with an average yield of 3.92 t/ha [4]. Myanmar has diverse landscapes and geographic variation ranging from the delta area of the Ayeyarwady River in the southern region to the mountainous areas in the north. This landscape heterogeneity resulted in the diversification of rice production systems, such as deep-water fields in the delta areas, irrigated and rainfed paddy fields in plain areas, and slash-and-burn fields in the mountainous areas. Geographic and crop diversity coupled with diverse traditional agricultural systems contribute to the high diversity of crop genetic resources in Myanmar [5,6].

Rice is mostly grown in the Ayeyarwady region, where it spreads through the deltaic watershed of the Ayeyarwady River, the longest river in Myanmar, which flows from the northernmost snowcap of the Himalayan Mountain range to the southern Ayeyarwady delta, ultimately draining into the Bay of Bengal. The richness of fertile alluvial soil and abundant monsoon rainfall in this delta provide vast fertile farmland. These attributes have defined the Ayeyarwady region as the largest “rice bowl” in Myanmar [7]. The total paddy area in the Ayeyarwady region covers approximately 2 million ha, which is 28% of the total cultivated rice area in Myanmar, and it produces 7.8 million t [4]. However, this region is highly vulnerable to the impacts of climatic aberrations evident from frequent saltwater intrusion and flooding. To tolerate such stresses, some unique rice landraces have been nurtured and cultivated by the farmers in this region over five decades. According to the survey results of Thant et al. [8], 66% of the farmers grow traditional rice varieties on their farmland, mainly during the rainy season, which indicates the high importance of the local varieties to counter the harsh environmental conditions. Myanmar has five categories of rice grain types based on length/width ratio: Emata (>3.3 mm), Latywezin (2.8–3.3 mm), Ngasein (2.4–2.8 mm), Meedon (2.0–2.4 mm), and Byat (2.25–3 mm) [9]. Among the most commonly grown varieties in the Ayeyarwady delta are the Pawsan varieties, belonging to the Meedon group, also called bold grain type. In addition, the genetic similarity of the Pawsan group was closer to japonica type than indica type [10]. This group has aroma, grain quality, and eating quality similar to those of the reputable aromatic rice varieties of the world: Basmati of India and Pakistan and Jasmine of Thailand. Pawsan rice is known as “Myanmar pearl rice” in the world market and received an award as the world’s best rice in 2011. Therefore, Pawsan varieties have special economic importance on the local market [11].

Local genetic resources (approximately 7000 genotypes including Pawsan rice) are conserved in the seed bank at the Department of Agricultural Research, Ministry of Agriculture, Livestock and Irrigation (MoALI), Yezin, Myanmar [12]. They represent the main source for genetic improvement for suites of traits, including tolerance of biotic and abiotic stresses. Moreover, identifying the available genetic diversity of local varieties compared with improved or introduced varieties could assist in developing the breeding strategy to design elite varieties for sustainable agriculture [13]. However, in Myanmar, unfortunately, only a small percentage of the available rice genetic resources have been used in past breeding programs. To overcome this bottleneck due to poor or scant information on the genetic characterization of available resources, our study could be a game changer for the development of Myanmar’s future rice breeding strategy that involves local diverse resources of paramount importance for climate resilience.

Nowadays, molecular markers to analyze genetic relationships in crops have become increasingly popular since they are more reliable than other phenotypic or biochemical markers [14,15]. Most studies up to now were conducted on limited sets of resources, using an older generation of markers such as restriction fragment length polymorphism (RFLP), amplified fragment length polymorphism (AFLP) and cleaved amplified polymorphic sequence (CAPS) [16], which are seldom used now because of poor marker-trait association information and, only in the recent past, have simple sequence repeat (SSR) markers been used [10,12,17,18,19,20]. SSRs and single nucleotide polymorphisms (SNPs) are the most common DNA markers for genetic studies [21]. According to a review article on microsatellite markers (SSR) and their application in *Oryza sativa* L. in Myanmar, SSR markers have been used most extensively to study the genetic diversity of more than 600 Myanmar rice landraces [22], while there is no report on using SNP markers to date. SNPs, biallelic markers, are excellent for genomic studies, particularly for marker-trait association, genomic selection, and determining population structure, because those studies require a high number of markers [23].

DArT (diversity array technology) markers developed by Jaccoud et al. [24] are useful for whole-genome profiling of crops without the need for prior sequence knowledge. This unique genotyping tool is characterized by hybridization rather than electrophoretic gel resolution and this helps to improve both throughput and accuracy. It can produce thousands of polymorphic loci in a single assay. DArT has generated two types of markers, SNP and silicoDArT, over the past decade. DArTseq-based SNPs are codominant markers. SilicoDArT markers are microarray markers that are dominant and scored for the presence or absence of a single allele. Compared to other marker technology, DArT markers have merits in terms of cost effectiveness and time [25]. There are only a few studies about genetic structure and diversity in rice using DArTseq markers [13,26], although many studies have been made on other crops such as barley (*Hordeum vulgare*), rye (*Secale cereale*), bean (*Phaseolus vulgaris*), macadamia (*Macadamia integrifolia*), etc. [27].

In view of this, we conducted genetic diversity and structure analyses involving a collection of currently cultivated rice germplasm accessions from farmers’ fields in addition to the collections from local gene banks using the most robust set of markers to obtain repeatable inferences. Thant et al. [8] extensively surveyed on-farm rice diversity in the Ayeyarwady region and found that the farmers in the survey area grow three main groups of rice: (i) Pawsan traditional varieties, (ii) traditional varieties other than Pawsan, and (iii) modern high-yielding varieties (HYVs). Therefore, we hypothesized the presence of population genetic structure with respect to the three groups collected in this panel of rice genotypes. The major objectives of this study were to (i) examine the genetic diversity and population structure and (ii) investigate genetic differentiation among and within populations of Myanmar rice varieties in the Ayeyarwady region using DArTseq technology.

## 2. Results

### 2.1. Marker Quality Analysis

Out of 18,271 SNP markers, a total of 7643 markers cleared and passed all the quality parameters (>95% reproducibility, >95% call rate, and >0.1 one ratio) (Figure S1). Among the 7643 informative SNPs, 43% were observed in the PIC class > 0.45 to 0.50 and 41% in the >0.30 to ≤0.45 class (Figure S2). The median (0.44) was located close to the average PIC value of 0.41 (Table S1). A total of 16,160 silicoDArT markers were generated and they had an average of 99% reproducibility and 93% call rate and 87% of all the identified markers had a >0.1 average one ratio (Table S2 and Figure S1). Considering all of the quality parameters, 4064 silicoDArT markers were used for subsequent analysis. These markers were determined to be moderately informative, with an average PIC value of 0.37 and 0.41 median (Table S1). Approximately 28% of the markers had a PIC value of ≤0.30 and 23% were in the high PIC value range (>0.45 to 0.50) (Figure S2). 

### 2.2. Genetic Relationships among Genotypes

**Figure 1:**
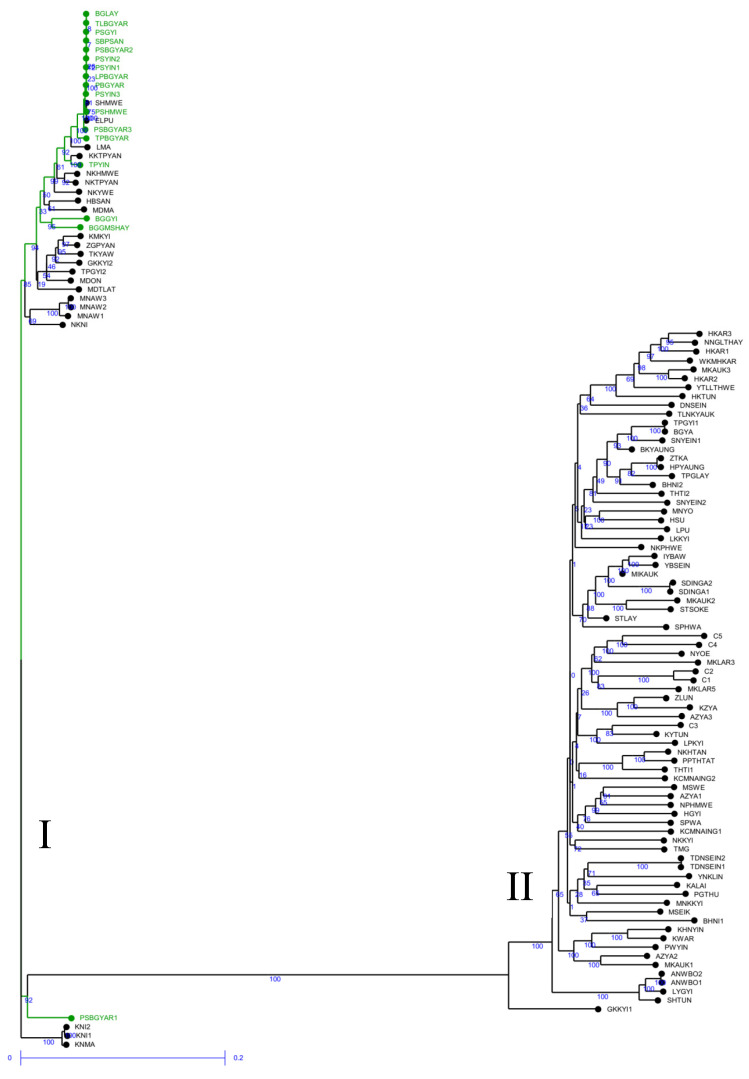
The weighted neighbor-joining phylogenetic tree based on 7643 SNP markers representing the grouping of 117 rice genotypes; Pawsan varieties are shown with green color.

The genetic dissimilarities among the genotypes estimated through the SNP markers ranged from 0 to 0.753 (Table S3). The Pawsan group of varieties revealed the least amount of genetic dissimilarity, ranging from 0 to 0.115, whereas the HYVs ranged from 0.037 to 0.217. Among the traditional varieties other than Pawsan, the dissimilarity indices ranged from 0 to 0.753. The weighted neighbor-joining phylogenetic tree obtained with SNP markers produced two major clusters (Figure 1). Cluster I consisted of 17 high-quality aromatic rice varieties (Pawsan group), while the remaining 23 genotypes were traditional varieties, which fell within the genetic dissimilarity range of 0 to 0.13 (Table S3), revealing that those varieties were closely related to the Pawsan group. Cluster II had a combination of traditional varieties and HYVs. Among the traditional varieties, NYOE, MKLAR3, MKLAR5, ZLUN, KZYA, AZYA3, KYTUN, and LPKYI displayed closer genetic similarity (dissimilarity indices of 0.11 to 0.233) to HYVs (Figure 1 and Table S3). 

**Figure 2:**
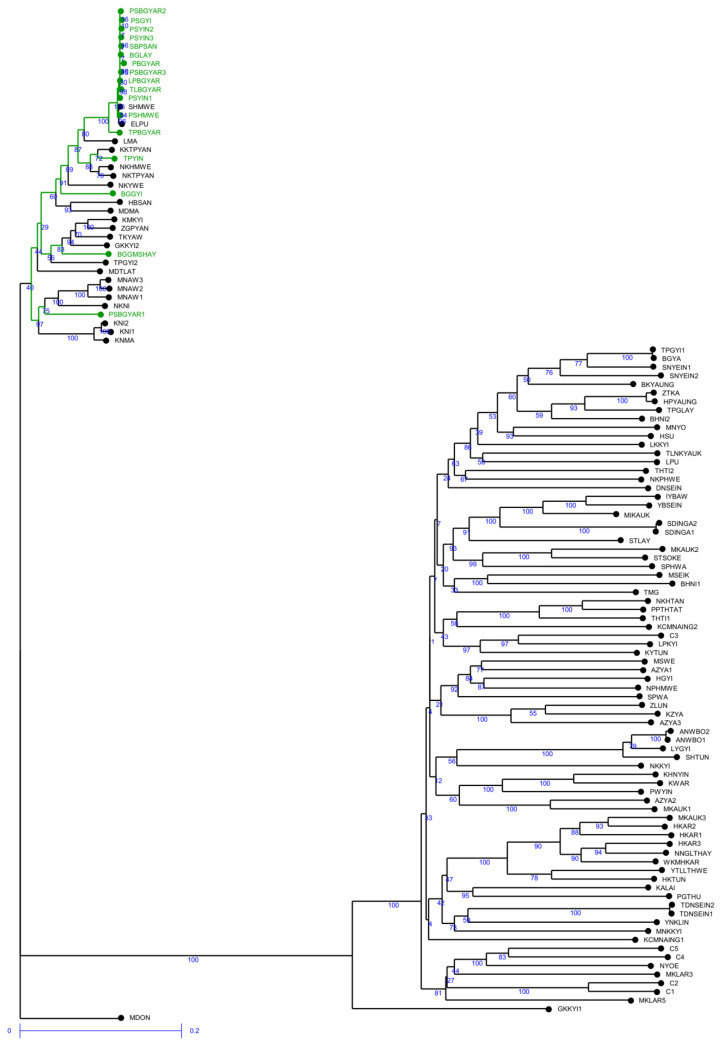
The weighted neighbor-joining phylogenetic tree based on 4064 silicoDArT markers representing the grouping of 117 rice genotypes; Pawsan varieties are shown with green color.

SilicoDArT markers were also useful for the identification of genetic relationships among rice genotypes. The range of genetic dissimilarities identified through silicoDArT markers was broader than that observed through SNP markers. Among the 117 rice genotypes, dissimilarity ranged from 0.001 to 0.954 (Table S4). The genetic dissimilarity index among Pawsan varieties fell within the range of 0 to 0.223, suggesting that they were closely related to each other. The HYVs ranged from 0.169 to 0.582, whereas the rest of the traditional varieties revealed a wide dissimilarity range, from 0 to 0.952. Similar to SNP markers, silicoDArT markers also formed two clusters of rice genotypes based on their relatedness (Figure 2). The proportion of membership of individual genotypes in each cluster showed consistency in grouping with the results of SNP markers.

### 2.3. Population Structure

**Figure 3:**
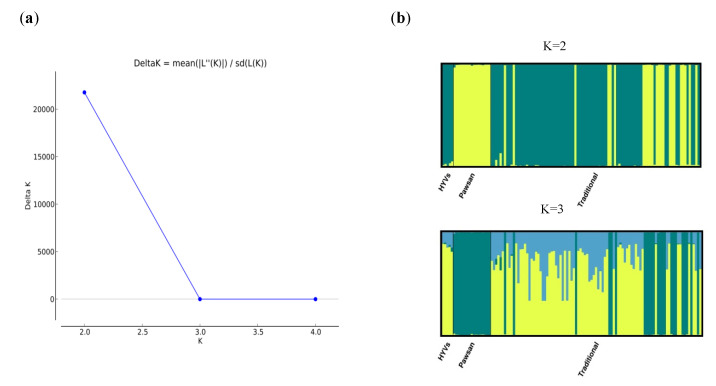
Population structure of 117 rice genotypes based on 7643 SNPs: (**a**) ΔK values plotted as the number of populations, (**b**) populations (K = 2 and K = 3) inferred using STRUCTURE. We observed the number of individuals at K = 3 as our hypothesis although there is no peak at K = 3. Traditional varieties, Pawsan varieties, and HYVs are colored differently.

A total of 7643 SNP markers were used for population structure analysis. The model-based Bayesian cluster analysis in STRUCTURE visualized the genetic structure of the population under examination. K value was used to estimate the number of clusters of the genotypes based on the genotypic data throughout the whole genome. In order to find the optimal K value, the number of clusters (K) was plotted against ΔK, which showed a sharp peak at K = 2 (Figure 3), and the membership of individual genotypes in each population is listed in Table S5. The optimal K value indicates that two populations showed the highest probability for population clustering and these two populations consisted of 40 (Pawsan plus non-Pawsan traditional varieties) and 77 (non-Pawsan plus HYVs), respectively (Table 1 and Table S5). 

**Figure 4:**
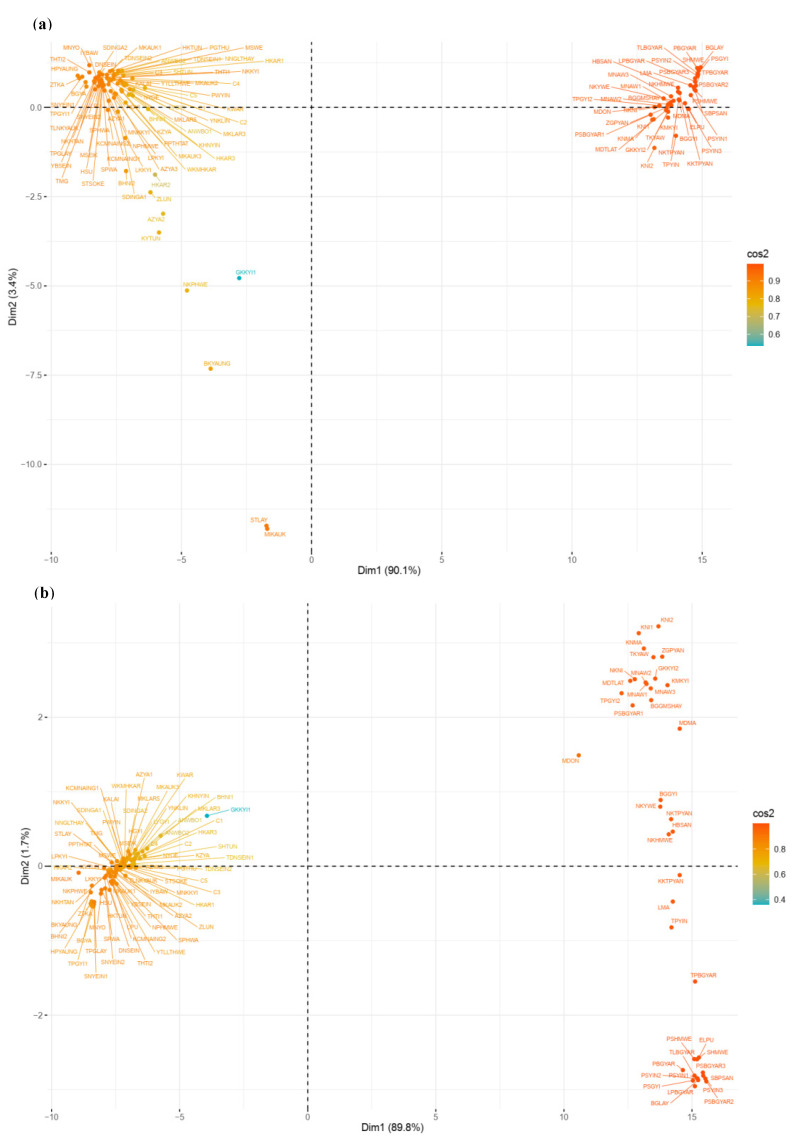
Principal component analysis (PCA) to explain the genetic diversity across 117 rice genotypes: (**a**) PCA based on 7643 SNP markers and (**b**) PCA based on 4064 silicoDArT markers.

A significant divergence was found among individuals within populations according to the Fst values of 0.832 for pop1 and 0.687 for pop2 obtained from STRUCTURE (Table 1). Principal component analysis (PCA) illustrated the genetic divergence among the genotypes (Figure 4). In SNP and silicoDArT markers, the first two axes of the PCA explained 93.5% and 91.5% of the total genetic divergence, respectively. The population distribution determined by both markers is consistent with the output of population structure analysis (Figure 4).

### 2.4. Genetic Differentiation of Populations

Results from the analysis of molecular variance (AMOVA) revealed that 4% of the total variance was found within individuals, whereas maximum diversity was partitioned between the two populations (74%) and among individuals within populations (22%) (Table 2). In addition, a high Fst (0.737) from the AMOVA results was found between populations, indicating a high genetic differentiation between these two populations, and a low Nm value (0.089) was obtained according to Nei’s genetic distance analysis (Table 2).

### 2.5. Allelic Pattern across Populations

The average value for the number of different alleles (Na) and effective alleles (Ne) across the populations was 1.604 and 1.252, respectively (Table 3), and the mean value for the overall population in Shannon’s index (I), expected heterozygosity (He), and unbiased expected heterozygosity (uHe) was 0.232, 0.150, and 0.152, respectively. Of the two populations, pop2 was more diverse than pop1 and the percentage of polymorphic loci per population (PPL) ranged from 41.07% (pop1) to 79.73% (pop2), with an average of 60.40%.

## 3. Discussion

### 3.1. Marker Quality Analysis

Our study highlights the suitability of DArT platforms that can be applied for genomic studies of rice genotypes. A total of 18,271 DArTseq SNPs were developed, of which 7643 markers provided robust information from the rice genome in the absence of sequence information, while silicoDArT markers provided 4064 informative markers. 

The average PIC values of both types of markers in rice were similar to the values identified in DArT markers in sorghum (0.41) [28], cassava (0.42) [29], and wheat (0.44) [30]. The PIC values are a good indication of informative markers that can be used for genotyping plant populations and studying genetic diversity [31]. According to a previous study, (1) markers with a PIC value of ≥0.50 were considered to be highly informative, (2) markers with a PIC value from 0.25 to 0.50 were moderately informative, and (3) markers with a PIC value of less than 0.25 were slightly informative [32]. The average PIC values of both SNP and silicoDArT markers in our results suggested that those markers were moderately informative. Marker density has a high correlation with gene density; therefore, the abundance of SNP and silicoDArT markers may achieve better genome coverage through the sampling of a greater number of points in the whole genome [33,34]. 

Of the different types of molecular markers, microsatellite markers (SSRs) have been used most extensively in Myanmar rice genotypes [12,17,18]. Mogga et al. [13] used DArTseq markers to investigate genetic diversity in rice (*Oryza sativa* L.). Their study was performed using 59 rice genotypes with 525 SNPs derived from a DArTseq platform. Phung et al. [35] also characterized a panel of 182 rice genotypes with 25,971 markers using DArT and SNP markers. Therefore, SNP and silicoDArT markers may be better suited for genetic diversity studies, association/linkage mapping, and sequence-based physical mapping in rice [27]. Furthermore, our study will be useful for international trade to avoid adulteration of Myanmar Pawsan varieties, which are priced varieties. Markers specific for identifying the sub-sub group of Pawsan could be helpful in identifying true Pawsan. This is similar to Basmati trade, in which international trading checks actual Basmati varieties through Basmati-specific markers.

### 3.2. Population Structure and Relationships

Population structure analysis is informative in understanding genetic diversity and facilitates subsequent association mapping studies [36]. We expected the presence of structure in this population to have three groups based on the findings of an on-farm rice diversity survey in the Ayeyarwady region [8]. However, the population structure results in our study did not support our hypothesis. In fact, 117 rice genotypes were divided into only two groups with STRUCTURE (K = 2) (Table 1). The dendrogram analysis (neighbor-joining tree) and the PCA results were in agreement with STRUCTURE results (Figure 1, Figure 2, Figure 3 and Figure 4). 

In total, 23 non-Pawsan traditional varieties and all 17 Pawsan varieties were clustered together into one genetically related population (pop1), which indicated that those non-Pawsan varieties were genetically close to Pawsan varieties. With regard to genetic dissimilarity indices, they revealed a smaller range (0 to 0.133) for SNP markers (Table S3) and a larger range (0 to 0.287) for silicoDArT markers (Table S4). All 40 genotypes clustered in pop1 had bold grain shape, which is called Meedon type in Myanmar. Most of the varieties in the Meedon group are local rice with good eating quality [20]. We assumed that those Pawsan and non-Pawsan rice varieties possessed the same genes that controlled rice grain shape. In addition, in the studies of Wunna et al. [12] and Thein et al. [20] using SSR markers, four genotypes (KMKYI, NKYWE, NKTPYAN, and ZGPYAN) out of 23 non-Pawsans varieties clustered together with Pawsan varieties. Thein et al. [10] studied the variation in genetic structure of 38 Pawsan rice varieties and reported that the Pawsan group was separate from two controls (IR36 for indica and Koshihikari for japonica). However, the genetic similarity of the Pawsan group was closer to japonica type (Koshihikari) suggesting that they were tropical japonica or javanica-type landraces. A former deputy director general of the Department of Agricultural Research also confirmed that the Pawsan group belongs to tropical japonica type varieties [37]. Therefore, we assumed that those 23 non-Pawsan varieties might also be tropical japonica types. In addition, morphological characterization on this panel of rice genotypes studied by Thant [38] pointed out that some traits, particularly culm length (91–120 cm), panicle length (~25 cm), and grain type (2–2.4 mm) were observed as common traits among genotypes within pop1. 

Further, 72 non-Pawsan traditional varieties and all 5 HYVs were clustered together in the second genetically related population (pop2), probably reflecting the fact that breeding activities led to genetic similarities since new varieties were usually selected from local landraces. Most of the local landraces have unique taste and shape, which breeders want to keep intact, whereas breeding for traits such as short duration, short stem, low canopy type, etc. However, local varieties have some undesirable traits such as long duration, tall or plant architecture etc. Although these varieties fell into the same group, they had a broad range of dissimilarity: 0–0.281 and 0.001–0.688 for SNP and silicoDArT markers, respectively (Tables S3 and S4). Moreover, Khush et al. [39] reported that indica-type landraces predominate in Myanmar (81% of the total landraces). Some genotypes in pop2 such as NPHMWE, KHNYIN, HKAR, C1, and C3 were confirmed as indica type [40]. It might be important that those 77 genotypes (non-Pawsan and HYVs) were found in the same population although further studies such as adding more control indica-type genotypes would be helpful to clarify this. 

We also observed the assignment of individuals to each population at K = 3 as our hypothesis (Figure 3b). The membership and number of genotypes (40) comprising pop1 at K = 3 were identical to the results at K = 2, whereas 77 genotypes in pop2 at K = 2 were divided into two groups (Table S6). This clearly indicates that there is no genetic similarity between Pawsan varieties and HYVs. Pawsan is known as a highly photoperiod-sensitive variety and its grain quality (a prized trait) depends upon photosensitivity and requires ecology (special growing season, etc.). For this reason, rice breeders/researchers failed to develop HYVs from the Pawsan group and consumers also refuse to accept any change from the original Pawsan varieties. Seven traditional varieties (other than Pawsan) out of 77 genotypes (72 non-Pawsan and 5 HYVs) found separation as a new group (pop3), suggesting that the genetic relatedness of those 7 non-Pawsan varieties with HYVs was less than with other traditional varieties grouped together with HYVs in pop2 (Table S6). Moreover, in the work of Thant [38] characterizing the same set of genotypes phenotypically, some traits (e.g., grain type, culm length, and number of spikelets per panicle) distinguished seven non-Pawsan traditional varieties from HYVs.

In addition, our study found that some duplicate genotypes, by their name (farmer-named varieties based on farmers’ taxonomies and nomenclature), were consistent with the naming and distinguishing through very close genetic relationships (e.g., in pop1: MNAW1, MNAW2, and MNAW3) (Figure 1). On the other hand, different genotypes accounted for by the same name also existed in this germplasm collection since the genotypes under the same name were classified into different groups, for instance, GKKYI1 (pop2) and GKKYI2 (pop1) (Figure 1). In this case, the combined use of molecular markers and morphological characters may allow further correct discrimination. Therefore, testing the underlying population structure is crucial for rice improvement strategies involving marker-trait association studies, such as genome-wide association scanning to identify a true association between markers and traits and the underlying genes controlling the traits [36]. The information obtained from such testing will build confidence in the outcome of the potential association that may be detected.

### 3.3. Genetic Differentiation of Populations

Fst (fixation index) is a measure of population differentiation due to genetic structure. A Fst value of 0.25 can be considered as significant in differentiating populations. The range 0.15–0.25 indicates moderate differentiation, whereas differentiation is negligible if the Fst value is 0.05 or less [41]. A significant divergence was found among individuals within populations according to the Fst values obtained from STRUCTURE (Table 1). The AMOVA results showed that high genetic variation existed between populations, which may be due to low genetic exchange and gene flow (Nm value) [42]. An Nm value of less than 1 indicates limited gene exchange among populations [43]. In our study, the Nm value (0.089) was quite low; therefore, the low genetic exchange between the two populations led to their high genetic differentiation [36]. Various cultural and agro-ecological factors influence the mechanisms of gene flow in rice fields [44]. In our study, the main factor influencing gene flow may be agro-ecological conditions. For instance, farmers whose fields were located near the sea (saline-water region) usually grew local varieties such as LYGYI, ANWBO1, and ANWBO2 because of their tolerance of salinity, whereas there was no possibility to grow the Pawsan group in such areas. This might have influenced the high genetic variation observed. 

The allelic pattern and genetic diversity indices provided useful information on the genetic diversity in each population. The higher value for diversity indices is an indication of a higher level of genetic diversity [36,45]. Previous studies in Myanmar rice using SSR markers observed a higher level of polymorphism with respect to heterozygosity [20,40] because of their multi-allelic nature and their rapid mutation rates [46]. SNPs are mostly bi-allelic; however, a higher number of loci sufficiently polymorphic can potentially give a similar genetic resolution as randomly chosen and multi-allelic SSRs [47]. In our study, comparing genetic diversity indices revealed that pop2 appeared to be more diverse with higher values for private alleles, Shannon’s index, expected heterozygosity, unbiased expected heterozygosity, and parental percentage of polymeric polymorphic loci. The level of diversity represents a valuable resource for future rice improvement programs. In the study of Thant et al. [8] on farmers’ preferences for rice varieties in the Ayeyarwady region, except for high yield, farmers were interested in rice varieties with a good response to stress conditions and suitability in particular agro-ecological regions, specifically those with salinity, submergence, and pests/diseases. Among the genotypes in pop2, some of them have those properties; for example, HKAR is resistant to nematode (*Ditylenchus angustus*) attacks, rice blast disease (*Magnaporthe oryzae*), and rice stem borer (*Scirpophaga incertulas*) [8]; MKAUK, HKAR, and MKLAR are well known for their submergence tolerance and elongation ability; and LYGYI and ANWBO are known for their salinity tolerance. Thus, our findings based on whole-genome genotyping could be a pillar for region-specific rice improvement programs that could meet local farmers’ demand.

## 4. Materials and Methods

### 4.1. Plant Materials

**Figure 5:**
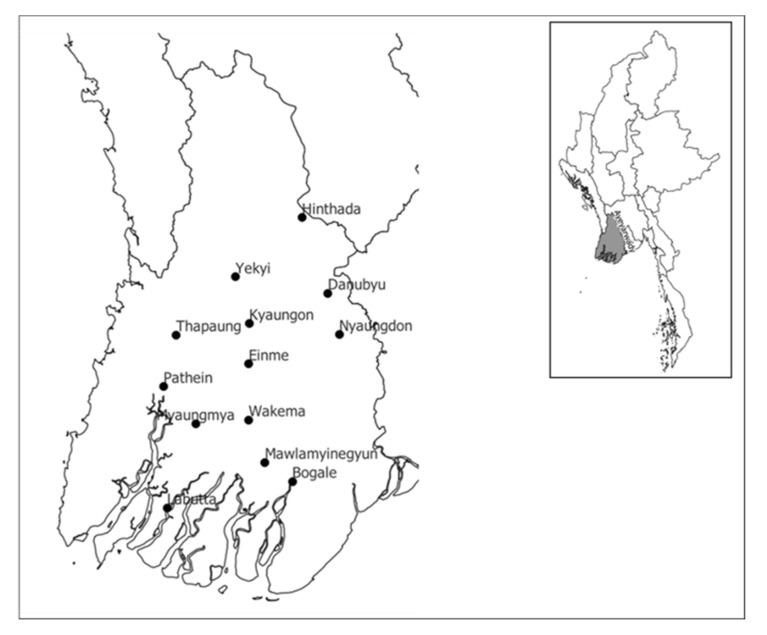
Area-showing collection sites for 112 rice genotypes in the Ayeyarwady delta.

In our study, we used a total of 117 rice genotypes, which included 95 traditional varieties (other than Pawsan), 17 traditional Pawsan, and 5 HYVs (Table S7). All genotypes except the HYVs were originally derived from the Ayeyarwady region (Figure 5). Seventy-two genotypes were provided by the Department of Agricultural Research (DAR) seed bank section in Yezin and 40 were directly collected from farmers’ fields in different parts of the Ayeyarwady region. Five popular HYVs, which can be grown across the country, were obtained from the International Rice Research Institute (IRRI-DAR), Yezin, and they were used as controls (check varieties).

### 4.2. DNA Extraction and Genotyping

Young healthy leaf tissue was collected from 3-week-old rice plants and stored on silica gel in plastic tubes for desiccation. DNA was isolated from dried leaves using the CTAB method developed by Doyle and Doyle [48] and modified by Faleiro et al. [49]. The content and purity of DNA were measured on a Nanodrop (Thermo Scientific, Waltham, MA, USA) spectrophotometer. DNA quality of the samples was controlled by incubating 1 µL of DNA in restriction enzyme buffer at 37 °C for 2 h and resolving the DNA on a 0.8% agarose gel in 1x TAE buffer. The DNA concentration was adjusted within the range of 50–100 ηg/μL. 

A total of 20 μL of each sample with a concentration of 100 ng/μL DNA were sent to Diversity Arrays Technology (DArT) Pty. Ltd., Bruce, Australia (https://www.diversityarrays.com, accessed on 19 November 2021) for whole-genome scans using a combination of DArT complexity reduction method and next-generation sequencing platform. Whole-genome genotyping for the 117 rice genotypes was carried out using DArTseq technology as described by Barilli et al. [50] using 18,271 SNPs and 16,160 silicoDArT markers (Tables S2 and S8). This method involved the digestion of DNA samples with a rare cutting enzyme, PstI, paired with a set of secondary frequently cutting restriction endonucleases, ligation with site-specific adapters, and amplification of adapter-ligated fragments. The DNA samples were processed in digestion/ligation reactions according to the prescribed standard procedures of Kilian et al. [34] but replacing a single PstI-compatible adapter with two different adapters corresponding to two different restriction enzymes overhangs. The genomic representations were generated following the procedures described by Kilian et al. [34] and Barilli et al. [50]. PstI-MseI was selected as the appropriate complexity reduction method for rice and consequently next-generation sequencing technology using HiSeq2500 (Illumina, San Diego, CA, USA) was employed to detect SNPs and silicoDArT markers. The sequence data were analyzed using DArTsoft14 and silicoDArT (presence/absence of markers in genomic representations) were scored ‘1’ for presence, and ‘0’ for absence.

### 4.3. Marker Quality Analysis

The following parameters for the DArT marker assaying pipeline for quality control were used for marker screening: reproducibility (%), call rate (%), polymorphism information content (PIC), and one ratio [34]. A total of 18,271 SNP and 16,160 silicoDArT markers were reported, of which 7643 SNP and 4064 silicoDArT markers were considered for analyses after filtering with quality control parameters including >95% reproducibility, >95% call rate, and >0.1 one ratio. Scoring of reproducibility involved the proportion of technical replicate assay pairs for which the marker score exhibited consistency. The call rate determined the success of reading the marker sequence across the samples and was estimated from the percentage of samples for which the score was either 0 or 1. PIC is the degree of diversity of the marker in the population and it showed the usefulness of the marker for linkage analysis. One ratio constitutes the proportion of the samples for which genotype scores equaled 1 [27].

### 4.4. Genetic Relationships among Genotypes and Population Structure

Genetic dissimilarity matrices were constructed in DARwin v. 6.0.21 to identify the genetic relationships among the genotypes [51]. Weighted neighbor-joining dendrograms were constructed in both marker (7643 SNP and 4064 silicoDArT) platforms. Clade strength in the dendrograms was tested by 10,000 bootstrap analyses. Principal component analysis for 7643 SNP and 4064 silicoDArT markers was conducted by R package factoextra [52]. Genetic structure using STRUCTURE v.2.3.4 among 117 rice genotypes using 7643 DArTseq-derived SNP markers was investigated [53]. Bayesian clustering method was applied to identify clusters of genetically similar individuals using STRUCTURE. The parameters used were burn-in period of 50,000 steps followed by 100,000 Monte Carlo Markov Chain iterations, admixture model with correlated frequencies, K varying from 1 to 5, and three runs per K value in order to obtain consistent results. The log-likelihood of the observed data for each K value was calculated and compared across the range of K values. The best K value was estimated based on the membership coefficient (Q) for each individual in each cluster. The Q values indicate the level of relatedness of each genotype to various subgroups. The STRUCTURE results were subsequently analyzed by the STRUCTURE HARVESTER application to identify the best value of K [54]. 

### 4.5. Analysis of Molecular Variance (AMOVA) and Genetic Diversity Indices

The number of clusters determined with STRUCTURE using 7643 SNPs was used for AMOVA and the calculation of Nei’s genetic distance using GenAlEx v6.503 [55]. From AMOVA, the Fst (fixation index) and Nm (haploid number of migrants) within the population were obtained. In addition, genetic indices such as number of loci with private allele, number of different alleles (Na), number of effective alleles (Ne), Shannon’s information index (I), observed heterozygosity (Ho), and expected heterozygosity (He) were also calculated using GenAlEx v6.503 [55].

## 5. Conclusions

Our study used DArTseq technology (SNP and silicoDArT markers) to examine the genetic diversity and population structure of 117 Myanmar rice genotypes. SNP and silicoDArT markers developed a large number of highly polymorphic markers and were a robust and relatively inexpensive option for future allele/gene identification. To date, this study was the first to use DArT in genetic diversity analysis on Myanmar rice genotypes. Based on our findings, the rice panel was genetically diverse. This level of genetic diversity could be the basis for developing new rice varieties with desirable characteristics such as high yield potential, high eating quality, and tolerance of abiotic stresses while being adapted to diverse environments. Moreover, this study identified two populations that could be explained by regional adaptation and natural selection. The presence of structure in this studied rice panel did not meet our expectations, which were to have three populations. HYVs were not found as a separate group. Instead, they were clustered together with several non-Pawsan traditional varieties in pop2. This might be due to breeding activities, suggesting that some breeding activities led to genetic similarities since new varieties were selected from local/traditional varieties. We also investigated some non-Pawsan varieties that were closely related to Pawsan varieties, and they were grouped together in pop1. The pop2 has a larger number of genotypes and exhibited higher values for genetic diversity indices and was thus more diverse than pop1. These findings will be important for future genetic analyses, such as allele/gene identification using genome-wide association studies, which is an approach that can identify the most important alleles for grain quality. Additionally, this study is advantageous for international trade to avoid adulteration, which means that markers specific for identifying the sub-group of Pawsan varieties could be helpful in identifying true Pawsan, which are highly priced varieties. 

## Figures and Tables

**Table 1:** STRUCTURE results of 117 rice genotypes assigned to each population.

Population	Inferred Clusters	Mean Fst ^1^	Exp. Het. ^2^	No. of Genotypes
pop1 ^3^	0.449	0.832	0.128	40
pop2 ^4^	0.551	0.687	0.169	77

^1^ Fst, fixation index; ^2^ Exp. het., expected heterozygosity; ^3^ pop1, Pawsan plus non-Pawsan traditional varieties; ^4^ pop2, non-Pawsan plus HYVs.

**Table 2:** Analysis of molecular variance (AMOVA) using 7643 SNPs of the genetic vari ation among and within two populations of 117 rice genotypes.

Source	df	SS	MS	Est. Var.	%
Among populations	1	209,102.877	209,102.877	1973.534	74
Among individuals	115	148,509.820	1291.390	585.915	22
Within individuals	117	13,988.500	119.560	119.560	4
Total	233	371,601.197		2679.009	100
Fst ^1^	0.737 (P = 0.001)				
Nm ^2^	0.089				

^1^ Fst, fixation index; ^2^ Nm, haploid no. of migrants.

**Table 3:** Genetic diversity indices for the two population structures of 117 rice genotypes based on 7643 SNPs.

Pop	Na ^1^	Ne ^2^	I ^3^	Ho ^4^	He ^5^	uHe ^6^	F ^7^	PPL (%) ^8^
pop1	1.411	1.146	0.137	0.025	0.087	0.088	0.515	41.07
pop2	1.797	1.358	0.327	0.035	0.214	0.215	0.776	79.73
Mean	1.604	1.252	0.232	0.030	0.150	0.152	0.688	60.40

^1^ Na, number of different alleles; ^2^ Ne, number of effective alleles; ^3^ I, Shannon’s index; ^4^ Ho, observed heterozygosity; ^5^ He, expected heterozygosity; ^6^ uHe, unbiased expected heterozygosity; ^7^ F, fixation index; ^8^ PPL, percentage of polymorphic loci.

## Data Availability

The data presented in this study are available in the Supplementary Materials.

## Supplementary Materials

The following are available online at https://osf.io/fkm7a/?view_only=a8f688fd90fe43e9a566284e354929ad, Figure S1: Distribution of SNP and silicoDArT marker data for several quality parameters: (a) reproducibility, (b) call rate, and (c) one ratio, Figure S2: Distribution of PIC values of SNP and silicoDArT markers used for genomic studies in rice, Table S1: Summary statistics of informative markers of both markers of the DArT platform, Table S2: List of silicoDArT markers, Table S3: Dissimilarity indices among 117 Myanmar rice genotypes estimated by neighbor-joining analysis of 7643 DArTseq based SNP markers in DARwin software, Table S4: Dissimilarity indices among 117 Myanmar rice genotypes estimated by neighbor-joining analysis of 4064 silicoDArT markers in DARwin software, Table S5: A list of membership in two population groups, Table S6: A list of membership in three population groups, Table S7: 117 Myanmar rice genotypes and their collection locations, Table S8: List of SNP markers.

## Abbreviations

AFLP, Amplified Fragment Length Polymorphism; AMOVA, Analysis of Molecular Variance; CAPS, Cleaved Amplified Polymorphic Sequence; CTAB, Cetrimonium bromide/ Cetyltrimethylammonium bromide; DAR, Department of Agricultural Research; DArT, Diversity Array Technology; DARwin, Dissimilarity Analysis and Representation for windows; DNA, DeoxyriboNucleic Acid; HYV, High Yielding Variety; ha, hectare; IRRI, International Rice Research Institute; MoALI, Ministry of Agriculture, Livestock and Irrigation; PCA, Principal Component Analysis; PIC, Polymorphism Information Content; Q, membership coefficient; RFLP, Restriction Fragment Length Polymorphism; SNP, single nucleotide polymorphisms; SSR, simple sequence repeat; t, ton.
